# Vertebral artery ruptures manifesting as hoarseness^☆^

**DOI:** 10.1016/j.bjorl.2016.11.003

**Published:** 2016-12-13

**Authors:** Chih-Jen Yang, Sheng-Yao Cheng, Cheng-Chung Cheng, Chi-Tun Tang, Shih-Hung Tsai

**Affiliations:** a National Defense Medical Center, Tri-Service General Hospital, Department of Emergency Medicine, Taipei, Taiwan; b National Defense Medical Center, Tri-Service General Hospital, Department of Otorhinolaryngology – Head and Neck Surgery, Taipei, Taiwan; c National Defense Medical Center, Tri-Service General Hospital, Department of Internal Medicine, Taipei, Taiwan; d National Defense Medical Center, Tri-Service General Hospital, Department of Neurological Surgery, Taipei, Taiwan

## Introduction

A retropharyngeal hematoma forms when blood accumulates in the deep space of the neck – the retropharyngeal space. Traumatic retropharyngeal hematoma after a minor trauma without cervical spine displacement is a rare occurrence. Retropharyngeal hematoma can quickly become life threatening because of its risk of airway compromise and requires immediate assessment and treatment. We report a case of a 67 year-old man who developed fatal retropharyngeal hematoma following minor blunt trauma.

## Case report

**Figure 1:**
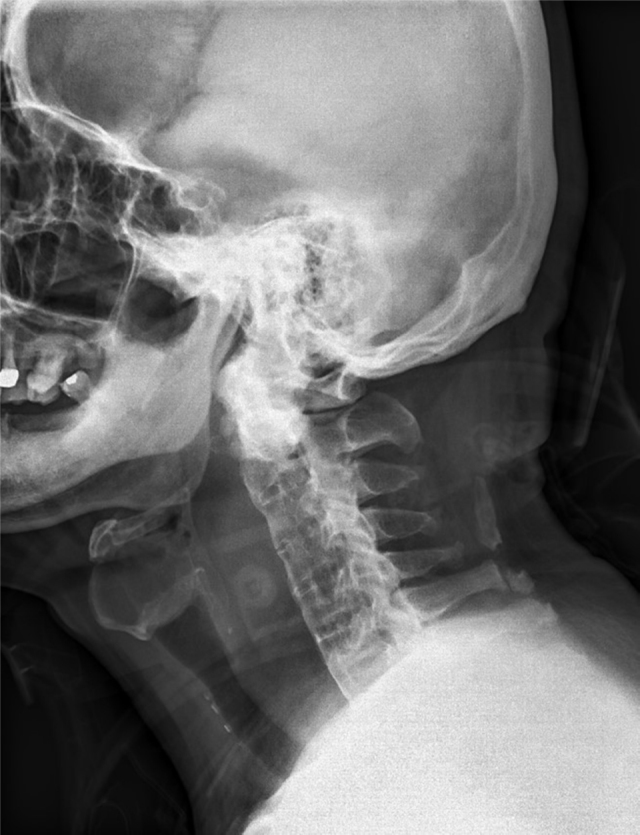
Lateral cervical plain radiogram showing widening retropharyngeal space (>7 mm at the C3 level).

**Figure 2:**
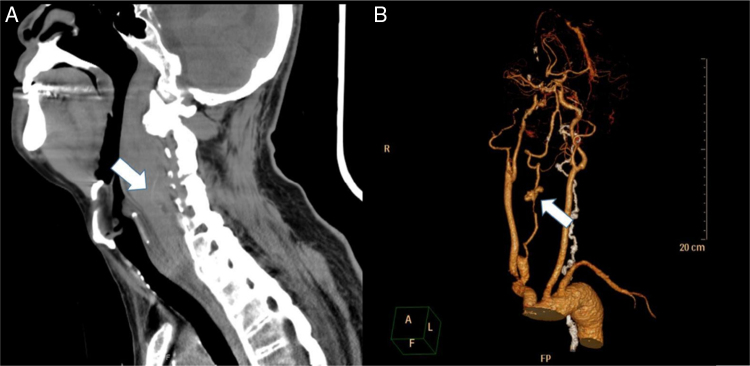
Contrast-enhanced computed tomography scan of the neck showing (A) the retropharyngeal hematoma extending from the base of the skull to the C7 level and syndesmophytes of the spine and (B) the pseudo-aneurysm formation on the right vertebral artery.

A 67-year-old man without significant medical history slipped on the stairs, fell, and hit his forehead on the floor. He was brought to our emergency department in an ambulance. On arrival, he was awake and alert with stable vital signs and complained of pain over his forehead. He denied initial loss of consciousness. Physical examination showed an abrasion wound about 3 cm × 2 cm with ecchymosis over the forehead; other findings were unremarkable. A non-contrast-enhanced computed tomography (CT) scan of the brain showed no significant findings. Laboratory tests (complete blood count, coagulation tests, serum aminotransferase, and creatinine levels) did not reveal any abnormality. During observation at the emergency department, the patient complained of sore throat and hoarseness. A lateral cervical plain radiogram was obtained, which demonstrated a widening retropharyngeal space (Fig. 1). Laryngoscopy showed bulging over the posterior pharyngeal wall, with narrowing space between the posterior pharyngeal wall and the epiglottis. Contrast-enhanced CT scan of the neck was performed, which revealed C4 fracture, syndesmophytes of the spine, 2.7 cm retropharyngeal hematoma, and pseudo-aneurysm formation on the right vertebral artery at the C4 vertebral level (Fig. 2). The patient developed dyspnea along with stridor shortly after the CT scan. He then received oroendotracheal intubation and was transferred to the intensive care unit.

**Figure 3:**
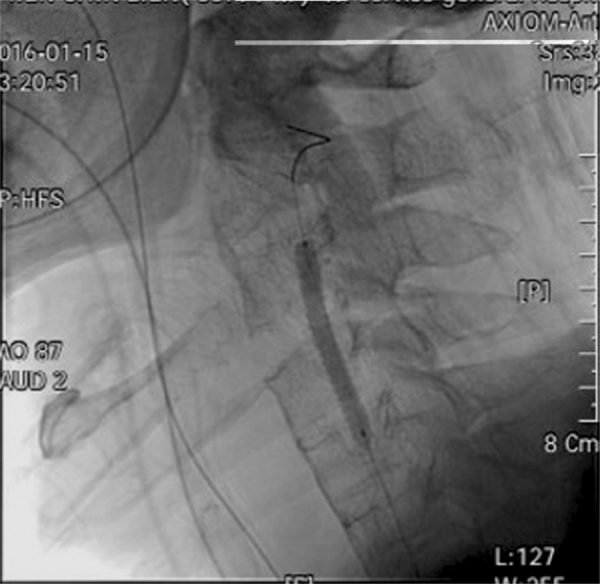
Angiography showing stent deployment over the right vertebral artery.

A cardiologist was consulted, and a 5.0 mm × 50 mm Viabahn stent was deployed at the right vertebral artery (Fig. 3). No neurological deficit was noted after stent implantation. The patient's recovery was uneventful. Further imaging and positive human leukocyte antigen B27 confirmed the presence of ankylosing spondylitis. He was transferred to the respiratory care center for extubation evaluation on hospital Day 8.

## Discussion

Retropharyngeal hematoma is thought to result mainly from the rupture of the vertebral arteries or its small branches or from the tearing of the anterior longitudinal ligament. Great vessels such as the thoracic aorta and thyrocervical trunk have also been reported.^1^, ^2^ Various mechanisms that may lead to traumatic retropharyngeal hematoma have been described: (1) hyperextension when falling on the head, car accidents, exercise including yoga, calisthenics, archery, and even painting a ceiling; (2) direct trauma to the pharyngeal wall by ingestion of foreign bodies, oral intubation, jugular vein cannulation, or cervical surgery. Predisposing factors include older age, coagulation disorders (or anticoagulant medication), vascular lesions, and preexisting vertebral bone deformities.^3^

As patient history can be nonspecific and the onset can be insidious, a high index of suspicion is needed to diagnose retropharyngeal hematoma even in a patient who presents several days after the initial insult. Patients with retropharyngeal hematoma can present with neck pain, torticollis, trismus, dysphonia, dysphagia, drooling, hemoptysis, or respiratory distress. On endoscopic inspection of the oral cavity, a mass may be visualized in the posterior pharyngeal wall of the oropharynx. A lateral cervical radiogram or a cervical CT image may reveal marked widening of the prevertebral space, confirming the clinical diagnosis of retropharyngeal hematoma.^4^ The upper limit of the normal thickness of the prevertebral space on a radiogram or CT scan is 7 mm at the C3 level.^5^ Usually, a CT scan is sufficient to make a diagnosis, but occasionally, magnetic resonance imaging is required to further differentiate blood from pus.

Establishing an airway is the initial focus in management. Opinions vary as to the optimal method of maintaining the airway. Some advocate endotracheal intubation by an experienced clinician, during which cervical spine injury must be assumed and the spinal cord protected. Others recommend immediate tracheostomy because they believe that it is the safest means of securing the airway while avoiding further damage to the posterior pharyngeal wall or rupture of the hematoma, which can add further injury to an already compromised airway.^2^, ^6^ However, rupture due to endotracheal intubation has not been reported.^7^

Once the airway is secured, the next main issue is hematoma management, which entails either drainage or observation. Surgical exploration and trans-oral aspiration have been tried, but neither showed any advantage over conservative treatment and entailed an increased risk of infection.^2^ Patients with small, nonexpanding hematoma can be treated conservatively with cervical spine immobilization. For hematomas that fail to regress or are rapidly expanding, drainage is indicated. In our case, the cause of the retropharyngeal hematoma was the right vertebral artery pseudo-aneurysm at the C4 level. We chose endovascular intervention rather than a surgical approach because open surgery is usually limited to very few specific cases, especially in the vertebral territory because it is deep and surrounded by many nervous structures.^8^, ^9^, ^10^ The patient's retropharyngeal hematoma turned out to be noninvasive and was successfully treated without further neurological complications.

Despite being caused by a minor injury, retropharyngeal hematoma can quickly become life threatening with airway compromise. In our case, the patient had concurrent ankylosing spondylitis, which might have also contributed to the development of such potentially lethal complication after a mild injury. Although hoarseness is only a “soft sign” of neck trauma, physicians should always consider the possibility that acute upper airway compromise may be caused by a retropharyngeal hematoma after minor head or neck injury and the patient must be given the highest triage priority.

## Conclusion

Retropharyngeal hematoma with life-threatening airway compromise requires rapid recognition and securing the airway. Endovascular intervention rather than a surgical approach to the hematoma in the vertebral territory should be considered.

## Conflicts of interest

The authors declare no conflicts of interest.

## References

[1] Kubota H., Endo H., Noma M. (2013). Airway obstruction by a retropharyngeal hematoma secondary to thoracic aortic aneurysm rupture. J Cardiothorac Surg.

[2] Van Velde R., Sars P.R., Olsman J.G., Van De Hoeven H. (2002). Traumatic retropharyngeal haematoma treated by embolization of the thyrocervical trunk. Eur J Emerg Med.

[3] Senthuran S., Lim S., Gunning K.E. (1999). Life-threatening airway obstruction caused by a retropharyngeal haematoma. Anaesthesia.

[4] Shiratori T., Hara K., Ando N. (2003). Acute airway obstruction secondary to retropharyngeal hematoma. J Anesth.

[5] Rojas C.A., Vermess D., Bertozzi J.C., Whitlow J., Guidi C., Martinez C.R. (2009). Normal thickness and appearance of the prevertebral soft tissues on multidetector CT. Am J Neuroradiol.

[6] Suzuki T., Imai H., Uchino M. (2004). Fatal retropharyngeal haematoma secondary to blunt trauma. Injury.

[7] Coleman J.A., Johnson J.T. (1986). Retropharyngeal hematoma: complication of cervical fracture. Otolaryngol Head Neck Surg.

[8] Inaraja Perez G.C., Rodriguez Morata A., Reyes Ortega J.P., Gomez Medialdea R., Cabezudo Garcia P. (2015). Endovascular treatment of a symptomatic vertebral artery pseudoaneurysm. Ann Vasc Surg.

[9] Mokin M., Dumont T.M., Kass-Hout T., Levy E.I. (2013). Carotid and vertebral artery disease. Prim Care.

[10] Li F., Song X., Liu C., Liu B., Zheng Y. (2014). Endovascular stent-graft treatment for a traumatic vertebrovertebral arteriovenous fistula with pseudoaneurysm. Ann Vasc Surg.

